# COVID-19 diagnosis using deep learning neural networks applied to CT images

**DOI:** 10.3389/frai.2022.919672

**Published:** 2022-08-05

**Authors:** Andronicus A. Akinyelu, Pieter Blignaut

**Affiliations:** Department of Computer Science and Informatics, University of the Free State, Bloemfontein, South Africa

**Keywords:** COVID-19 diagnosis, convolutional neural network, CT images, deep learning networks, pre-trained models

## Abstract

COVID-19, a deadly and highly contagious virus, caused the deaths of millions of individuals around the world. Early detection of the virus can reduce the virus transmission and fatality rate. Many deep learning (DL) based COVID-19 detection methods have been proposed, but most are trained on either small, incomplete, noisy, or imbalanced datasets. Many are also trained on a small number of COVID-19 samples. This study tackles these concerns by introducing DL-based solutions for COVID-19 diagnosis using computerized tomography (CT) images and 12 cutting-edge DL pre-trained models with acceptable Top-1 accuracy. All the models are trained on 9,000 COVID-19 samples and 5,000 normal images, which is higher than the COVID-19 images used in most studies. In addition, while most of the research used X-ray images for training, this study used CT images. CT scans capture blood arteries, bones, and soft tissues more effectively than X-Ray. The proposed techniques were evaluated, and the results show that NASNetLarge produced the best classification accuracy, followed by InceptionResNetV2 and DenseNet169. The three models achieved an accuracy of 99.86, 99.79, and 99.71%, respectively. Moreover, DenseNet121 and VGG16 achieved the best sensitivity, while InceptionV3 and InceptionResNetV2 achieved the best specificity. DenseNet121 and VGG16 attained a sensitivity of 99.94%, while InceptionV3 and InceptionResNetV2 achieved a specificity of 100%. The models are compared to those designed in three existing studies, and they produce better results. The results show that deep neural networks have the potential for computer-assisted COVID-19 diagnosis. We hope this study will be valuable in improving the decisions and accuracy of medical practitioners when diagnosing COVID-19. This study will assist future researchers in minimizing the repetition of analysis and identifying the ideal network for their tasks.

## Introduction

Humans have been victims of various pandemics throughout history, with a few proving disastrous. The globe is dealing with another deadly and highly contagious virus—COVID-19. This virus was reported in Wuhan, China, on December 31, 2019 (Zhu et al., 2020), and on March 11, 2020, the World Health Organization (WHO) declared it a pandemic. According to Johns Hopkins University database, the COVID-19 pandemic has infected over 543 million people and killed over 6.3 million since June 2022. Moreover, more than 185 countries have reported cases of COVID-19. On November 24, 2021, a new and highly contagious strain of the COVID-19 virus, called the Corona Omicron variant, was identified. The WHO noted that this strain spreads faster than any other COVID variant. There is an urgent need for techniques that can effectively tackle COVID-19 and reduce its spread significantly.

Many techniques have been proposed in the literature for COVID-19 diagnosis. Ardakani et al. (2020) proposed a DL-based approach for COVID-19 detection. They trained ten Convolutional Neural Network (CNN)-based pre-trained models on 1020 Computed Tomography (CT) slices from 108 patients. The results showed that ResNet101 and Xception produced the best results, achieving an accuracy of 99.51 and 99.02%, respectively. Shi et al. (2020) proposed a machine learning framework for COVID-19 classification. They used decision trees to classify subjects into two groups based on the size of infected lesions. They also used Random Forest to classify each group as COVID-19 or pneumonia patients. They evaluated the technique on chest CT images, and it produced an F1-Score of 0.91. Song et al. (2021) introduced a DL-based system for CT diagnosis. They evaluated the technique on 88 COVID-19 infected CT images and 86 CT images from healthy individuals. The method achieved an Area Under Curve (AUC) of 0.99. According to the authors, the technique can extract main lesion features, particularly the ground-glass opacity, which is helpful for doctors.

Ozturk et al. (2020) developed a DL technique for early detection of COVID-19 cases using chest X-ray images and a DarkNet model. The method was designed to diagnose binary and multi-class classification. The technique was evaluated on 1,125 chest X-ray images consisting of 125 COVID-19 images, 500 no-findings images, and 500 pneumonia images. The results show that the method produced a classification accuracy of 98.08% for binary classes and 87.02% for multi-class classification. Loey et al. (2020) presented a hybrid technique for coronavirus diagnosis in chest X-ray images using a Generative Adversarial Network (GAN) and deep transfer learning. They used a dataset containing 69 COVID-19 images, 79 normal images, and 158 pneumonia images. Moreover, they used GAN to increase the size of the dataset to 8,100 images. Different experiments were performed on three DL models, and the results show that Googlenet produced an accuracy of 80.6% for four-class classification, AlexNet produced an accuracy of 85.2% for three-class classification, and Googlenet achieved an accuracy of 100% for binary classification.

Li et al. (2020) developed a DL model for COVID-19 diagnosis using CT images. They trained the model on 400 COVID-19 CT images, 1,396 community-acquired pneumonia images, and 1,173 non-pneumonia CT images. The results showed that ResNet50 produced a sensitivity and specificity of 90 and 96%, respectively. Horry et al. (2020) proposed a semi-automated DL technique for COVID-19 detection on X-ray images. They developed a new approach for reducing unwanted noise from X-ray images based on the GrabCut algorithm (Rother et al., 2004). They evaluated their model on 400 X-ray images (100 COVID-19 images) and five DL pre-trained models, namely VGG16, VGG19, InceptionV3, Xception, and Resnet50. The results showed that VGG19 achieved the best precision of 83%. Albahli and Albattah (2020) developed DL-based COVID-19 classification techniques using three pre-trained models, namely Inception-ResNetV2, InceptionNet-V3, and NASNetLarge. They fine-tuned the models on X-ray images, and the results show that the InceptionNet-V3 model produced the best classification accuracy of 98.63% (with data augmentation) and 99.02% (without data augmentation).

This study presents deep learning (DL) based solutions for COVID-19 diagnosis using CT images and 12 cutting-edge DL pre-trained models with acceptable Top-1 accuracy. This study is different from other studies in the following ways:

1. This study presents the results and performance analyses of 12 pre-trained, state-of-the-art CNN-based models for COVID-19 diagnosis using CT images. Twelve distinct CNN-based pre-trained models were trained from end to end using the original images in an open-source COVID-19 dataset, and the same 12 models were trained using the transformed images of the COVID-19 dataset. This study presents the results and performance analysis of the original and transformed datasets.
2. As shown in Table 1, most of the previously published studies examined one to five pre-trained CNN models, except for (Vijayaakshmi, 2019; Ardakani et al., 2020), which evaluated eight and ten pre-trained models, respectively. This study analyses the performance of twelve CNN pre-trained network architectures, which is more than the number of architectures examined in previous studies. In addition, most researchers train their models using X-ray images, while this study used CT images. CT images capture blood vessels, bones, and soft tissues more effectively than X-Ray images (Vijayaakshmi, 2019).
3. As indicated in Table 1, the dataset used in most studies contains a small number of COVID-19 images (between 60 and 600 COVID-19 images). The dataset used in this study has 9,000 COVID-19 images and 5,000 normal images from 1,537 patients. The dataset was used to train the 12 models examined in this study. Experimental results show that most of the models correctly classified the COVID-19 and non-COVID-19 images in the dataset with high confidence. Moreover, the models are compared with those in three existing studies, and they outperformed the compared techniques.

**Table 1:** Dataset information from previous studies.

**Name**	**Image type**	**Dataset information**	**# of patients**	**# of pre-trained models**
Ardakani et al. (2020)	CT scan	510 COVID-19, 510 Non-COVID-19	108	10
Horry et al. (2020)	X-Ray	100 COVID-19, 200 Normal, 100 Pneumonia		5
He et al. (2020)	CT scan	349 COVID, 397 Non-COVID	216	8
Shi et al. (2020)	CT images	–	2,585	–
Song et al. (2021)	CT images	88 COVID-19, 86 Non-COVID	275	4
Ozturk et al. (2020)	X-ray Images	125 COVID-19, 500 no-findings images, and 500 pneumonia	–	1
Loey et al. (2020)	X-ray images	69 COVID-19, 79 normal, 158 pneumonia	–	3
Li et al. (2020)	CT images	400 COVID-19, 1,396 community-acquired pneumonia, and 1,173 non-pneumonia.	3,322	1
Albahli and Albattah (2020)	X-Ray Images	850 COVID-19, 915 normal images, and 500 Pneumonia.	–	3
This study	CT Images	9,000 COVID-19, 5,000 non-COVID-19	1,537	12

## Methods

DL is an area of machine learning that focuses on techniques inspired by the brain neurons (Rong et al., 2019). DL is rapidly gaining prominence as a tool for image classification and object detection. The following state-of-the-art DL network architectures are considered in this study: VGG16, VGG19, Xception, ResNet50, ResNet101V2, DenseNet169, InceptionV3, NASNetLarge, DenseNet201, MobileNetV2, InceptionResNetV2, and DenseNet121. This section presents an overview of the 12 architectures.

### Overview of pre-trained models

VGG16 is a CNN architecture developed by the Visual Geometry Group at the University of Oxford. The network was instrumental in winning the 2014 ImageNet competition. It is widely regarded as one of the finest vision model architectures to date (Faisal et al., 2021). VGG16 comprises of thirteen convolutional layers of 3 × 3 filters with a stride of 1 (Simonyan and Zisserman, 2014). It uses same padding and max pool layer of a 2 × 2 filter with a stride of 2. It maintains this order of convolution and max pool layers throughout the architecture. Finally, it has two fully-connected layers and an output layer. The network was trained on 1.2 million images with 1,000 classes.

VGG19 is a deeper network compared to VGG16. It consists of 16 convolutional layers and three fully-connected layers. It was trained on 1.2 million images with 1,000 classes (Simonyan and Zisserman, 2014). MobileNet-V2 is a low-weight pre-trained model comprised of four convolution layers, sixteen inverted residual and linear bottleneck blocks, and a fully-connected layer (Sandler et al., 2018). In total, the network consists of 52 convolutional layers and one fully-connected layer. The network's primary design is based on inverted residual blocks. ResNet-50 (He et al., 2016a) and ResNet-101V2 (He et al., 2016b) are two ResNet variations that were trained on the ImageNet dataset. ResNet50 is a 50-layer network (48 Convolution layers, 1 MaxPool, and 1 Average Pool layer). ResNet-50 additionally includes a variety of residual blocks. ResNet101 is made up of 101 layers and 33 residual blocks. Xception is a CNN with 71 layers of depth developed by Chollet (2017). It begins with two convolution layers and progresses through depth-separable convolution layers, four convolution layers, and a fully-connected layer. The depth-wise separable convolution layers are the most important component of the network's architecture.

The Densenet-169 (Huang et al., 2017), DenseNet201 (Huang et al., 2017) and DenseNet121 (Huang et al., 2017) models are all versions of the DenseNet model, one of the most recent breakthroughs in neural network-based visual object recognition. DenseNet is relatively similar to ResNet with a few key distinctions. ResNet employs an additive method (+) for combining the previous layer's (identity) output with the future layer, whereas DenseNet concatenates (.) the previous layer's output with the future layer. Due to the longer path between the input and output layers, information vanishes before it reaches its destination. DenseNet was developed to address the vanishing gradient effect in high-level neural networks. DenseNet is comprises of one convolutional layer, six transition layers, ten dense block layers, and one classification layer. Each dense block comprises a variable number of convolutions of varying sizes. The primary distinction between Densenet-121, DenseNet169, and DenseNet201 is the model's depth, size, and accuracy. Densenet-121, DenseNet169, and DenseNet201 have a total of 121, 169 and, 201 layers, respectively. They are each 33, 57, and 80 MB in size. They each achieved a Top-1 accuracy of 0.750, 0.762, and 0.773, respectively.

InceptionV3 (Szegedy et al., 2016) is a well-known CNN architecture from the inception family with 48 layers. It employs label smoothing and an auxiliary classifier for regularization, and factorized 7 × 7 convolutions to minimize the number of parameters without compromising the network's efficiency. Additionally, batch normalization iactsas a regularizer between the auxiliary classifier and the fully-connected layer. InceptionResNetV2 (Szegedy et al., 2017) is a 164-layer CNN-based model pre-trained on over a million images from the ImageNet collection. The network leverages the advantages of the inception network while also incorporating residual connections. It uses residual connections to replace the filter concatenation stage in the Inception architecture. The results of (Szegedy et al., 2017) demonstrate that residual connections considerably improve inception network training.

NASNetLarge (Zoph et al., 2018) is a CNN architecture that obtained a top-1 82.7% accuracy on the imageNet dataset. It uses a reinforcement learning search method to find the best architecture configurations. It is made up of both reduced and normal cells. The normal cells are convolutional cells that return a two-dimensional feature map (Zoph et al., 2018). Reduction cells are convolution cells that return a feature map with a two-fold reduction in feature map and breadth (Zoph et al., 2018).

All the models used in this study were pre-trained on the ImageNet dataset (Szegedy et al., 2015). More information on the pre-trained models is provided in Table 2.

**Table 2:** Information on the pre-trained models used in this study.

**Model**	**Top-1 accuracy**	**Parameters**	**Depth**	**Input layer size**
VGG16	0.71	138,357,544	23	224 × 224
VGG19	0.71	143,667,240	26	224 × 224
Xception	0.79	22,910,480	126	224 × 224
ResNet50	0.75	25,636,712	50	224 × 224
ResNet101V2	0.76	25,613,800	101	224 × 224
DenseNet169	0.76	14,307,880	169	224 × 224
InceptionV3	0.78	23,851,784	159	224 × 224
NASNetLarge	0.83	88,949,818	–	224 × 224
DenseNet201	0.77	20,242,984	201	224 × 224
MobileNetV2	0.71	3,538,984	88	224 × 224
InceptionResNetV2	0.80	55,873,736	572	224 × 224
DenseNet121	0.75	8,062,504	121	224 × 224

### Implementation details

The CNN pre-trained models examined in this study were built using Keras, an open-source neural network library. The entire training procedure is divided into two phases: (i) hyper-parameter search phase and (ii) evaluation. Different models are assessed during the hyper-parameter search stage, and the model with the optimal hyper-parameter configuration is chosen. The selected model is passed to the evaluation phase for training, validation, and testing.

A Keras tuner function is used to find the best hyper-parameter configuration during the hyper-parameter search stage. There are four types of tuners in the Keras tuner function: hyperband, Sklearn, Bayesian Optimization, and random search tuners. The random search tuner is used in this study. The tuner requires a model-building function that allows the user to specify network designs and different ranges of hyper-parameter values. Various models are constructed iteratively during the search by invoking the model-building function. The function populates the search space with values from the user-specified range of hyper-parameters. The tuner works its way through the search space while recording the results for each network configuration. Following the search, the best model can be obtained and fine-tuned for *n* epochs, where *n* is a user-defined number. All the models in this study were subjected to hyper-parameter tuning. All the evaluated models are trained for *n* epochs during the hyper-parameter search, with *n* = 2 in this study. We retrieved the best model (which had previously been trained for two epochs) and trained it for an additional six epochs after the search. As a result, the total number of training epochs for the entire model is eight.

**Figure 1:**
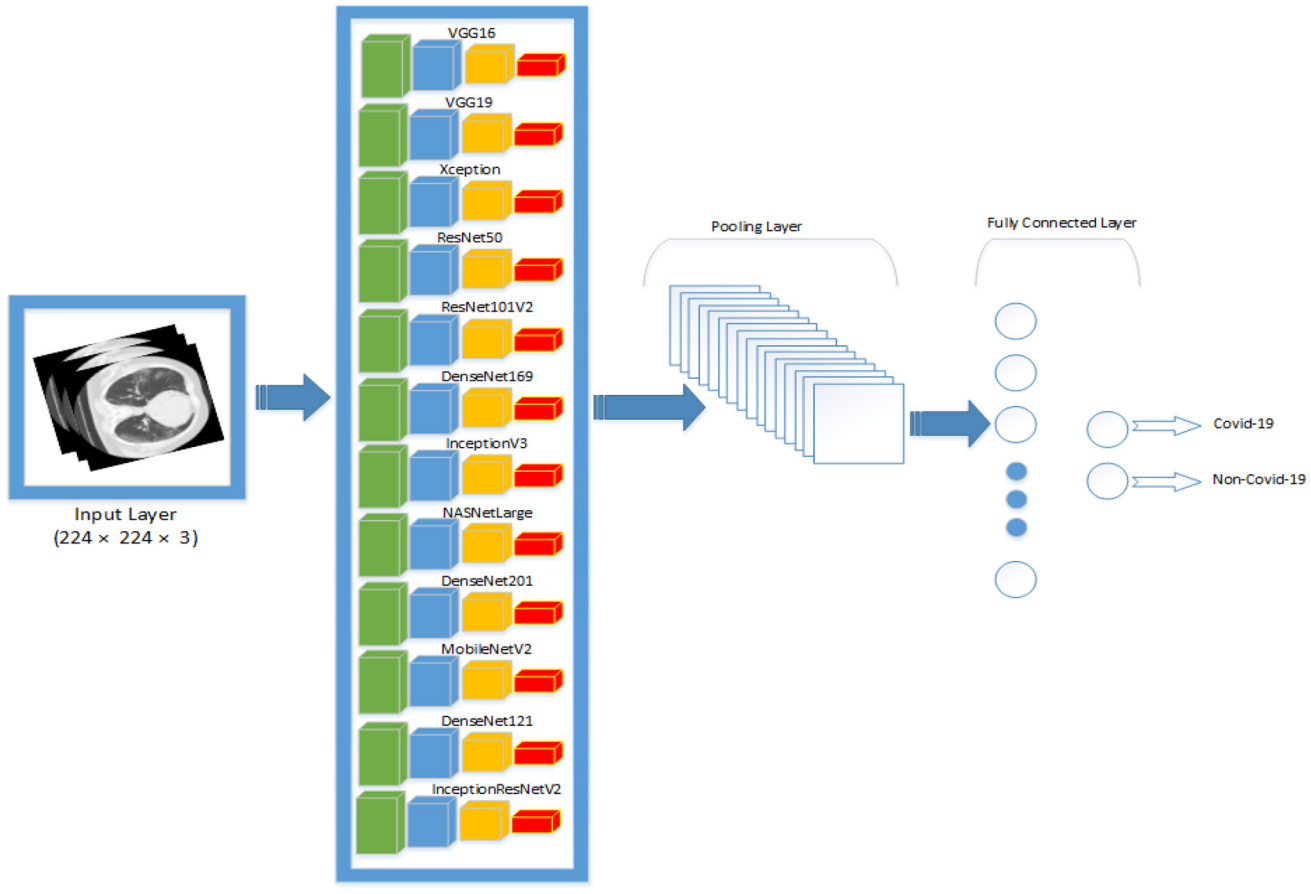
An overview of the network architecture that was used in this study. All the pre-trained networks are passed through one pooling layer, one fully-connected layer and one output layer.

Transfer learning was utilized to optimize the pre-trained models to the dataset. The output of the pre-trained models is passed through one average pooling layer, one dropout layer, and two fully-connected layers. Pooling layers are used to limit the number of parameters that must be learned and to enhance computation time. The dropout layer is used to avoid overfitting. The rate of dropout is set to 0.5. Each pre-trained model has a unique set of parameters. Various tests were conducted to ascertain the best number of hyper-parameters for each pre-trained model. For each model, the last fully connected layer has two neurons, one for each output (COVID-19 and non-COVID-19). Tables 3, 4 summarizes the hyper-parameters utilized in each model. During the studies, it was revealed that freezing all pre-trained layers prior to fine-tuning resulted in worse results than fine-tuning without freezing the pre-trained layers. As a result, all models in this study were fine-tuned without freezing their pre-trained layers. Figure 1 shows the network architecture that was employed in this study.

**Table 3:** Training parameters for the models without data augmentation.

**Model**	**Learning rate**	**Pooling layer**	**Fully-connected layer**
Xception	0.0001	3	80
VGG19	0.0001	5	96
ResNet50	0.0001	5	80
VGG16	0.0001	4	80
ResNet101V2	0.0001	5	112
DenseNet169	0.0001	5	112
InceptionV3	0.0001	5	112
NASNetLarge	0.0001	4	128
DenseNet201	0.0001	5	128
MobileNetV2	0.0001	4	96
InceptionResNetV2	0.0001	5	112
DenseNet121	0.0001	4	112

**Table 4:** Training parameters for the models with data augmentation.

**Model**	**Learning rate**	**Pooling layer**	**Fully connected layer**
Xception	0.0001	3	128
VGG19	0.0001	4	96
ResNet50	0.0001	3	96
VGG16	0.0001	5	96
ResNet101V2	0.001	3	112
DenseNet169	0.0001	4	80
InceptionV3	0.001	4	96
NASNetLarge	0.0001	4	80
DenseNet201	0.0001	5	112
MobileNetV2	0.0001	3	80
InceptionResNetV2	0.0001	3	96
DenseNet121	0.0001	3	112

Before training, the images in the dataset were resized to 224 × 224. Moreover, each image was normalized to the range [0, 1]. The images were normalized by dividing their pixel value by 255, the maximum pixel value. Finally, the images were used to train the model. Eighty percent of the dataset was used for training and twenty percent for testing. All the experiments were carried out on multiple nodes of a cluster computer. The cluster computer's specification is as follows: 2 x Intel Xeon E5-2697A v4 processors, 512 GB DDR4 memory running at 2.4 GHz. Each node is equipped with 32 cores and 64 threads. Each job submitted to the cluster used a maximum of three nodes, 20 GB of memory, and twenty cores.

### Performance evaluation

In this study, six performance measures were adopted, namely: accuracy, precision, sensitivity, specificity, F1 score, and duration.


$$\begin{array}{l} \text{Accuracy}=\frac{TN+\;TP}{TN+FN+TP+FP} \end{array}$$

Where TN, TP, FN, and FP refer to true negative, true positive, false negative, and false positive.


$$\begin{array}{l} \text{ Sensitivity}=\frac{TP}{TP+FN}\\ \text{ Specificity}=\frac{TN}{TN+FP}\\ \text{ Precision}=\frac{TP}{TP+FP}\\ \text{F1-Score}=\frac{2\;\times Precision^{*}Sensitivity}{Precision\;+Sensitivity} \end{array}$$

Duration = *T*_*tr*_+ *T*_*val*_+ *T*_*test*_, where *T*_*tr*_, *T*_*val*_, *T*_*test*_ refers to the total amount of time spent by each model during training, validation and testing, respectively.

### Datasets

The dataset used in this study consists of 194,922 CT images from an international cohort of 3,745 patients aged 0–93 years with findings that are clinically confirmed. The global cohort consists of patient records gathered from different sources worldwide, including the China National Center for Bio-information (Zhang et al., 2020), National Institutes of Health Intramural Targeted Anti-COVID-19 (An et al., 2020), Negin Radiology Medical Center (Rahimzadeh et al., 2021), Union Hospital and Liyuan Hospital of Huazhong University of Science and Technology (Ning et al., 2020), COVID-19 CT Lung and Infection Segmentation initiative (Ma et al., 2020) and Radiopaedia collection (Radiopaedia, 2021). The dataset was obtained from patients in the following countries: China, Iran, Italy, Turkey, Ukraine, Belgium, Australia, Afghanistan, Scotland, Lebanon, England, Algeria, Peru, and Azerbaijan. The dataset was carefully selected and processed by Gunraj et al. (2021). Moreover, the decision-making behavior of the dataset was reviewed by two certified radiologists with 10 and 30 years of experience (Gunraj et al., 2021). Validation was conducted to guarantee that decisions generated by models trained on the dataset are based on relevant visual indications in CT scans. Using correct, clinically relevant factors, the results shows that the decision-making behavior of the dataset is consistent with the interpretation of the radiologists (Gunraj et al., 2021).

**Figure 2:**
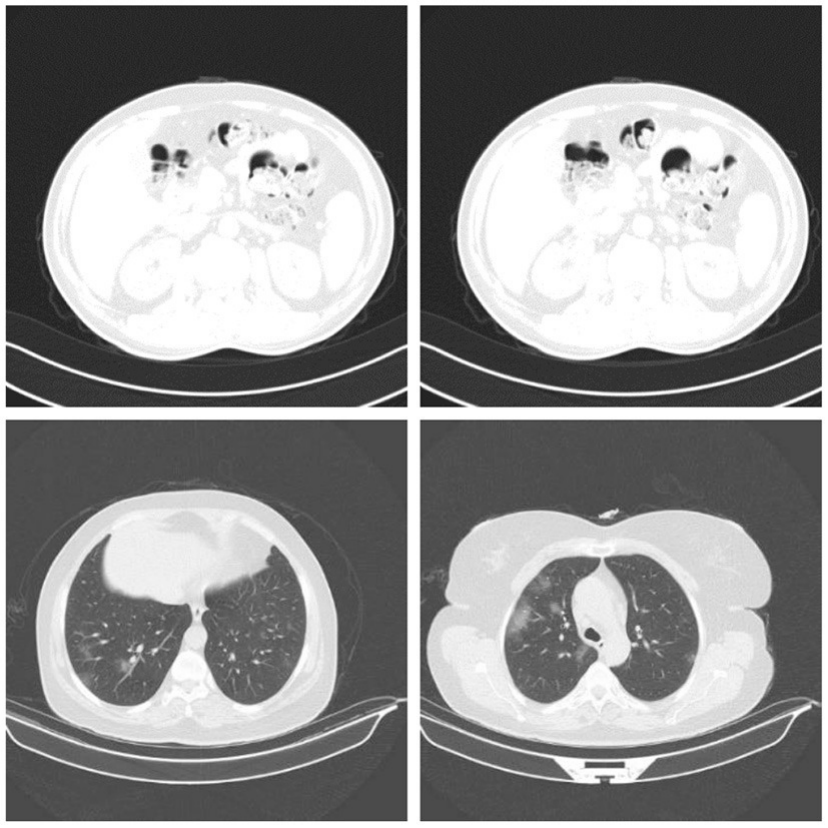
Samples of normal CT images (row 1) and COVID-19 CT images (row 2) used for evaluation (Gunraj et al., 2021).

In this study, we used a subset of the dataset. The subset consists of 14,000 CT images from 1,537 patients (9,000 COVID-19 CT images and 5,000 normal CT images). Kindly note that the CT images used in this study refers to CT slices of CT scans. The CT images (slices) are used as input to the pre-trained models. An example of normal CT images and COVID-19 CT images are shown in the first and second rows of Figure 2, respectively. During the experiment, the dataset was pre-processed to reduce the noise and to allow the DL models to focus exclusively on learning the critical features of the CT images. The pre-processing was performed using the OpenCV Grabcut algorithm (Rother et al., 2004). The GrabCut algorithm is an image segmentation technique that is based on graph cuts. The approach uses a Gaussian mixture model to predict the color distribution of the target object and the background. After processing the images with the GrabCut algorithm, we compared the quality of the processed images to the quality of the original images by building different models with both sets of images. The results showed that the original images produced better classification accuracy than the images processed with GrabCut algorithm. Therefore, for all our experiments, we used only the original images for training. Prior to training, the COVID-19 images in the dataset were labeled 1, and all other images were labeled 0. The images and their respective labels are used to build the COVID-19 models.

Table 1 provides the information of some datasets used in previous studies. As shown in the table, most of the datasets are small. In a bid to increase the size of a dataset, a researcher may want to combine different types of medical images [e.g., CT scans, Magnetic Resonance Image (MRI), X-Ray images], and use the combined dataset to build a model. This is not a common practice, as it can affect the CT system characteristics in the training pipeline. To address this, distinct models must be trained for each type of image. For example, the authors in Zopes et al. (2021) used MRI and CT scans to build a CNN model for brain scans. However, they did not use the MRI and CT scan to train the same model. They designed a common architecture and trained separate CNN models for each modality. In this study, data augmentation was used to increase the size of the training dataset. The results reported by Ramnarine (2021) indicate that image transformations can improve the performance of CNN models. Ramnarine (2021) applied four image transformations to a lungs cancer dataset, and the results showed that the image transformations considerably reduced the test loss of CNN model to half of the original test loss in some cases. Similarly, data augmentation was also applied to our COVID-19 dataset to improve the generalizability of the CNN models designed in this study. The following geometric transforms were applied to the dataset: random rotations, zooms, shifts, shearing, and horizontal flips. The specific values for the data augmentation are shown in Table 5.

**Table 5:** Data augmentation values.

**Rotation**	**Zoom**	**Width shift**	**Height shift**	**Shear**
**range**	**range**	**range**	**range**	**range**
0.2	0.15	0.2	0.2	0.15

## Discussion

In this study, 12 popular CNNs are used to examine the role of artificial intelligence in diagnosing COVID-19 infections. Different groups of experiments are performed. In the first group, 12 CNN pre-trained models were fine-tuned from end to end without data augmentation. The original images in the COVID-19 dataset were used to fine-tune the 12 models. In the second group of experiments, the 12 models were fine-tuned with data augmentation. The same 12 pre-trained models were fine-tuned using the transformed images of the COVID-19 dataset. After finetuning, the trained models were evaluated on the test dataset, and their result are presented in this section.

### Performance of pre-trained CNN architecture without data augmentation

**Figure 3:**
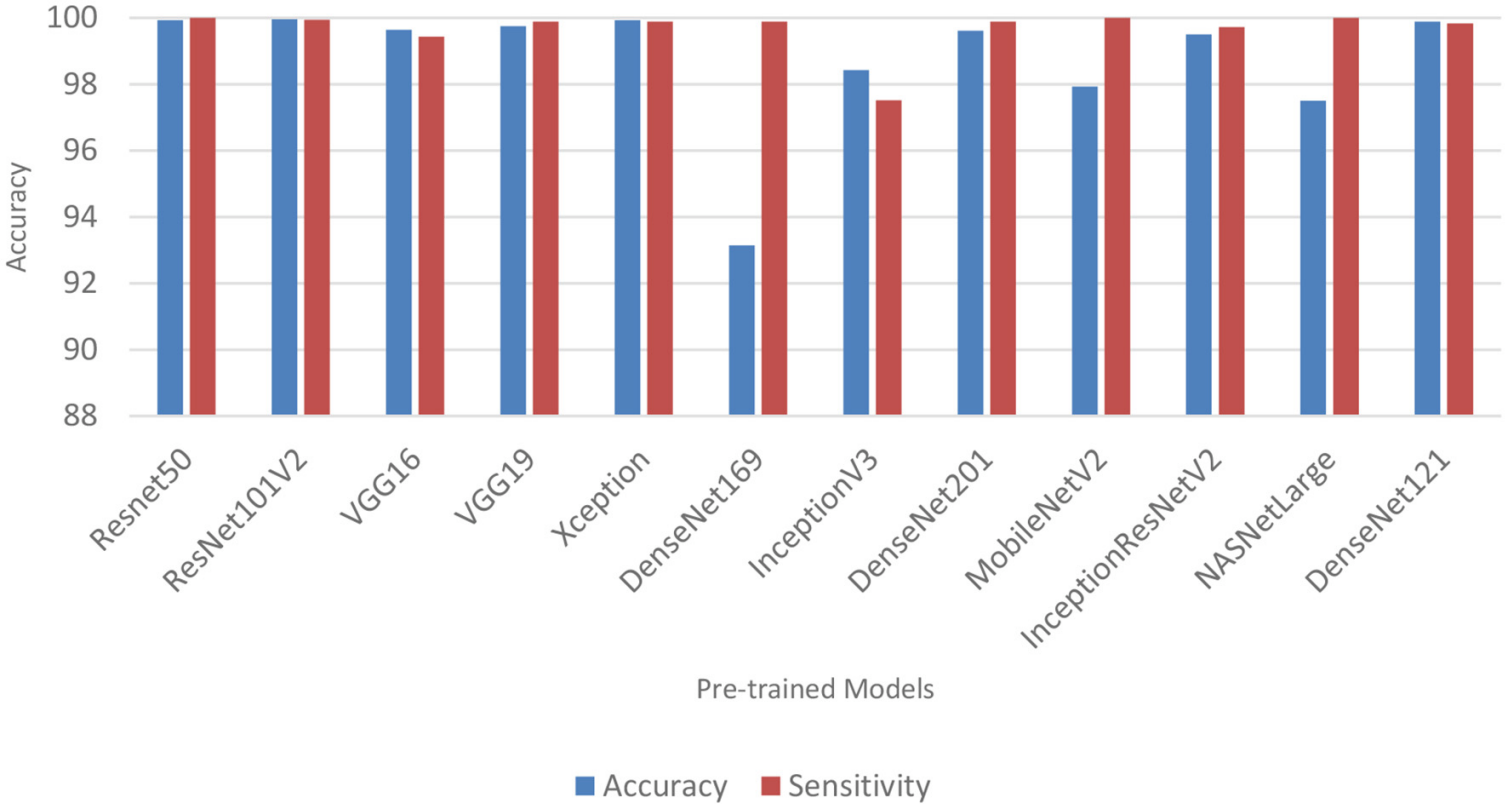
Classification accuracy and sensitivity of models without data augmentation.

Table 6 and Figure 3 shows the performance of the DL models without data augmentation. The numbers in bold represent the total number of true positive and true negative values. As can be seen, all the evaluated models achieved satisfactory performance indicating their effectiveness in diagnosing COVID-19 cases in CT images. In terms of classification accuracy, ResNet101V2, ResNet50, and Xception produced the best performance achieving an accuracy of 99.96, 99.93, and 99.93%, respectively. The other models achieved an accuracy of over 99% except for InceptionV3, MobileNetV2, and NASNetLarge, and DenseNet169. DenseNet169 produced the lowest classification accuracy of 93.14%. Moreover, all the models produced very good specificity, except for DenseNet169, NASNetLarge, and MobileNetV2. The three models produced a specificity of 81.52, 93.19, and 94.36%, respectively. This shows that most of the pre-trained models correctly identified over 99% of non-COVID-19 images. This is crucial, as it would not be desirable to diagnose healthy individuals as diseased.

**Table 6:** Results without data augmentation.

**Model Name**	**Accuracy**	**Precision**	**Sensitivity**	**Specificity**	**F1 score**	**Duration (hours)**	**Confusion matrix**		
							**C**	**N**	
Resnet50	99.93	99.89	100	99.81	1.00	4.55	**1,772**	2	C
							0	**1,026**	N
ResNet101V2	99.96	100	99.94	100	1.00	6.45	**1,771**	0	C
							1	**1,028**	N
VGG16	99.64	100	99.44	100	1.00	9.35	**1,762**	0	C
							10	**1,028**	N
VGG19	99.75	99.72	99.89	99.51	1.00	20.12	**1,770**	5	C
							2	**1,023**	N
Xception	99.93	100	99.89	100	1.00	6.32	**1,770**	0	C
							2	**1,028**	N
DenseNet169	93.14	90.31	99.89	81.52	0.95	8.78	**1,770**	190	C
							2	**838**	N
InceptionV3	98.43	100	97.52	100	1.00	3.68	**1,728**	0	C
							44	**1,028**	N
DenseNet201	99.607	99.494	99.887	99.12	1.00	14.6	**1,770**	9	C
							2	**1,019**	N
MobileNetV2	97.929	96.831	100	94.36	0.98	**2.73**	**1,772**	58	C
							0	**970**	N
InceptionResNetV2	99.500	99.493	99.718	99.12	1.00	5.98	**1,767**	9	C
							5	**1,019**	N
NASNetLarge	97.500	96.200	100	93.19	0.98	23.07	**1,772**	70	C
							0	**958**	N
DenseNet121	99.893	100	99.831	100	1.00	6.07	**1,769**	0	C
							3	**1,028**	N

*C, COVID-19 samples; N, Normal samples*.

Table 6 also shows the sensitivity achieved by each model. The results shows that ResNet50, MobileNetV2, and NASNetLarge diagnosed COVID-19 with the highest sensitivity. They all achieved a sensitivity of 100%. In the medical domain, developing a model with very high sensitivity is important. The high sensitivity of ResNet50, MobileNetV2, and NASNetLarge makes them a better fit for COVID-19 classification. It shows that the three models correctly identified most of the COVID-19 images. Their complex network structures and designs may be responsible for their improved performance. The design of ResNet50 is based on an ensemble of residual nets. The design of MobileNetV2 is based on inverted residual block and lightweight depth-wise convolutions. NASNetLarge uses a reinforcement learning search method to optimize architecture configurations. The residual connections, depth-wise convolutions, and reinforcement search is likely responsible for enhancing the training and optimization processes of the three models, resulting in a more reliable model.

Overall, the 12 pre-trained models achieved an accuracy, precision, sensitivity, and specificity of over 99%, except for DenseNet169, InceptionV3, MobileNetV2, and NASNetLarge. DenseNet169 produced the poorest accuracy, precision, and specificity, while InceptionV3 produced the poorest sensitivity. ResNet50, ResNet101V2, DenseNet121, and Xception produced the best overall result considering the models trained without data augmentation.

### Performance of pre-trained CNN architecture with data augmentation

**Figure 4:**
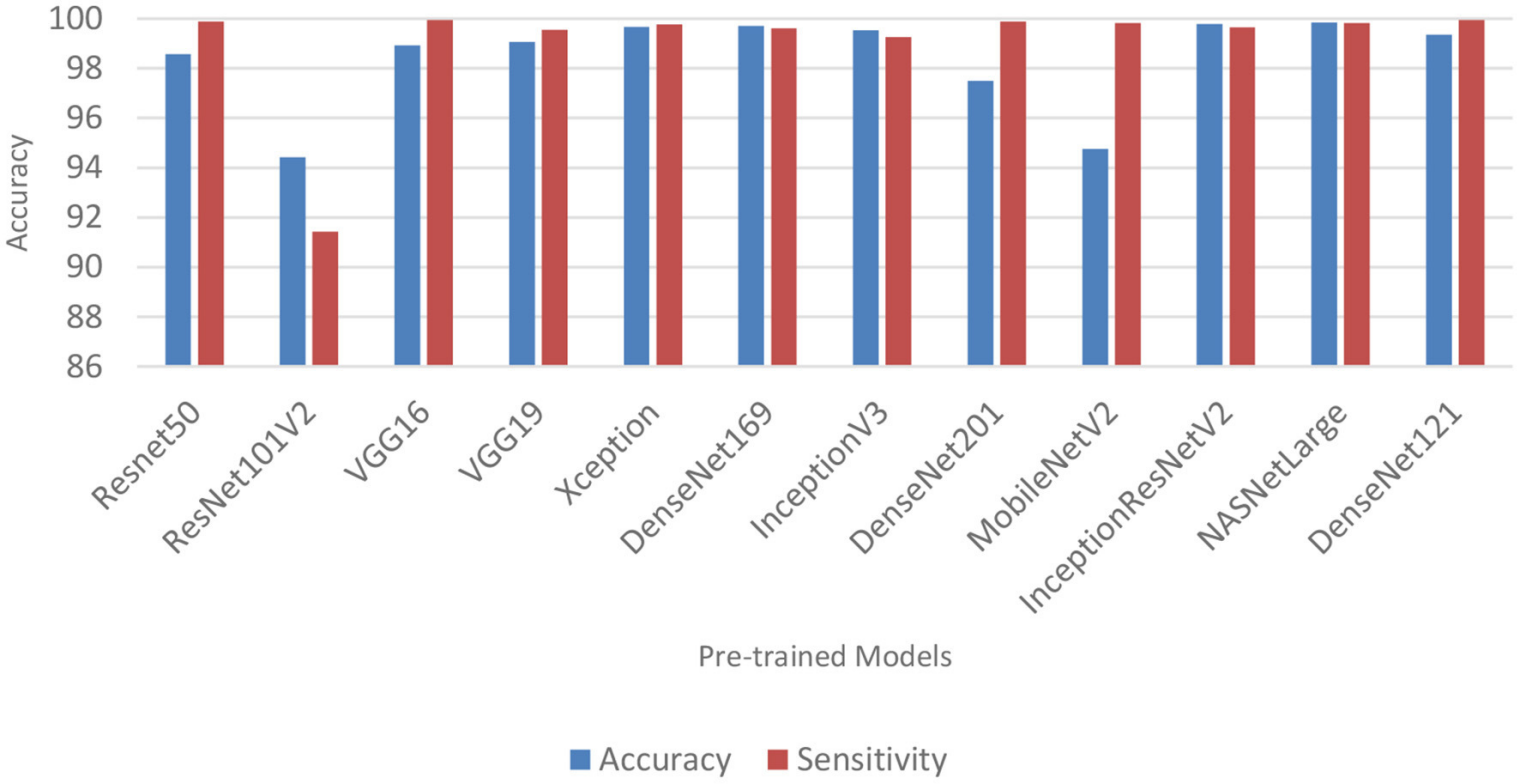
Classification accuracy and sensitivity of models with data augmentation.

Data augmentation was applied to the CNN inputs to increase their generalizability. Table 7 and Figure 4 shows the result of the models after the application of data augmentation. The numbers in bold represent the total number of true positive and true negative values. In terms of accuracy, NASNetLarge produced the best performance, followed by InceptionResNetV2 and DenseNet169. The three models achieved an accuracy of 99.86, 99.786, and 99.71%, respectively. In terms of sensitivity, DenseNet121 and VGG16 both achieved the best result, with both achieving a sensitivity of 99.944%. This shows that the two models correctly classified most of the COVID-19 images in the dataset with 99% confidence. The confidence score of a CNN model is very important, because the higher the score, the more confident the CNN model is that the prediction will satisfy the user. In most cases, we want our model to make the most accurate predictions of COVID-19 samples. A model that diagnoses a patient as COVID-19 negative when the patient is infected with the virus will be far more disastrous.

**Table 7:** Results after data augmentation.

**Model name**	**Accuracy**	**Precision**	**Sensitivity**	**Specificity**	**F1 score**	**Duration (hours)**	**Confusion matrix**		
							**C**	**N**	
Resnet50	98.57	97.90	99.89	96.30	0.99	8.68	**1,770**	38	C
							2	**990**	N
ResNet101V2	94.43	99.75	91.42	99.61	0.95	7.23	**1,620**	4	C
							152	**1,024**	N
VGG16	98.93	98.39	99.94	97.18	0.99	35.27	**1,771**	29	C
							1	**999**	N
VGG19	99.07	98.99	99.55	98.25	0.99	10.83	**1,764**	18	C
							8	**1,010**	N
Xception	99.68	99.72	99.77	99.51	1.00	6.40	**1,768**	5	C
							4	**1,023**	N
DenseNet169	99.71	99.94	99.61	99.90	1.00	11.57	**1,765**	1	C
							7	**1,027**	N
InceptionV3	99.54	100	99.27	100	1.00	3.45	**1,759**	0	C
							13	**1,028**	N
DenseNet201	97.50	96.30	99.89	93.39	0.98	23.35	**1,770**	68	C
							2	**960**	N
MobileNetV2	94.75	92.47	99.83	85.99	0.96	2.85	**1,769**	144	C
							3	**884**	N
InceptionResNetV2	99.79	100	99.66	100	1.00	6.75	**1,766**	0	C
							6	**1,028**	N
NASNetLarge	99.86	99.94	99.83	99.90	1.00	20.93	**1,769**	1	C
							3	**1,027**	N
DenseNet121	99.36	99.05	99.94	98.35	0.99	5.78	**1,771**	17	C
							1	**1,011**	N

*C, COVID-19 samples; N, Normal samples*.

**Figure 5:**
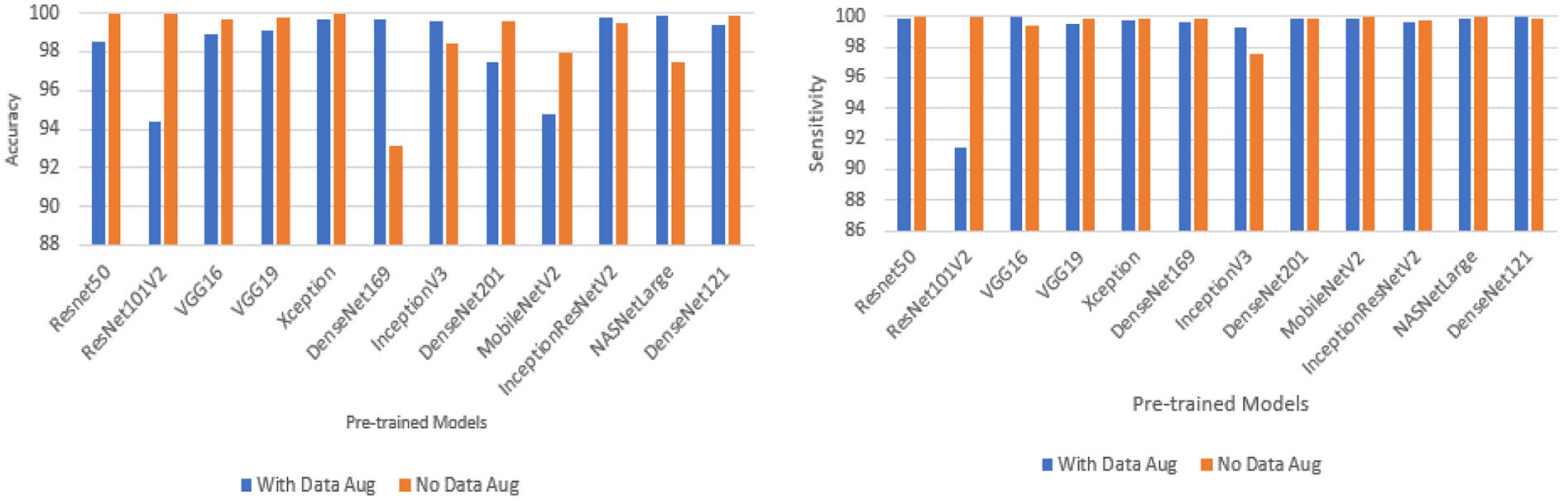
Comparison between models with data augmentation and models without data augmentation.

DenseNet201 and ResNet50 also produced very good sensitivity. Overall, as can be observed in Table 7 and Figure 5, the sensitivity of most of the models increased after data augmentation was applied. This shows the impact of image transformation in reducing overfitting and enhancing the model's ability to generalize or adapt to new, previously unknown data.

### Confusion matrix, network depth, parameter size, and time

Tables 6, 7 shows the confusion matrix for each model. The true positive and true negative values are highlighted in bold, while the false positive and false negative values are not highlighted. As can be seen for all the models, the number of correct predictions (TP and TN) is very high, while the number of incorrect predictions (FP and FN) is very low. This shows that most of the models predicted over 99% of the classes correctly, confirming their effectiveness in distinguishing COVID-19 CT scans from non-COVID-19 CT scans. Table 2 shows the parameter size and network depth of each architecture. The results shows that parameter size and network depth play a role in the performance of CNN models. As shown, even though MobileNetV2, DenseNet169, and Xception have the fewest parameters (3,538,984, 14,307,880, and 22,910,480 million, respectively), the three network architectures achieved one of the highest sensitivity values. Additionally, as demonstrated by the results, the depth of a network has a substantial effect on the effectiveness of DL networks in classifying COVID-19 cases. DenseNet169 and Xception have network depths of 169 and 126, respectively, and they achieved one of the best sensitivities. This demonstrates that deep networks outperform shallow network architectures in terms of classification performance.

The time required to train, validate, and test each model is summarized in Tables 6, 7. MobileNetV2 consumed the least time, followed by InceptionV3 and ResNet50. It is worth noting that MobileNetV2 consumed the least amount of time while still outperforming eight of the 12 models in terms of sensitivity. MobileNetV2 has a small number of parameters and a shallow depth, which makes it faster than the other models examined in this study.

### Model interpretation

Although, DL algorithms has produced very good results in computer vision tasks, one of the biggest challenges of DL is model interpretability, which is an important component in model understanding. Typically, DL models are treated as black box models, because we do not know how the network arrived at its final output, neither do we know the neurons that are activated during prediction. To handle this problem, Selvaraju et al. (2017) designed the Gradient-weighted Class Activation Mapping (Grad-CAM). The Grad-CAM uses the gradients of any target concept that flows to the final convolutional layer to produce localization maps (Selvaraju et al., 2017). The maps highlight the important regions and patterns in the image that were activated during the prediction.

**Figure 6:**
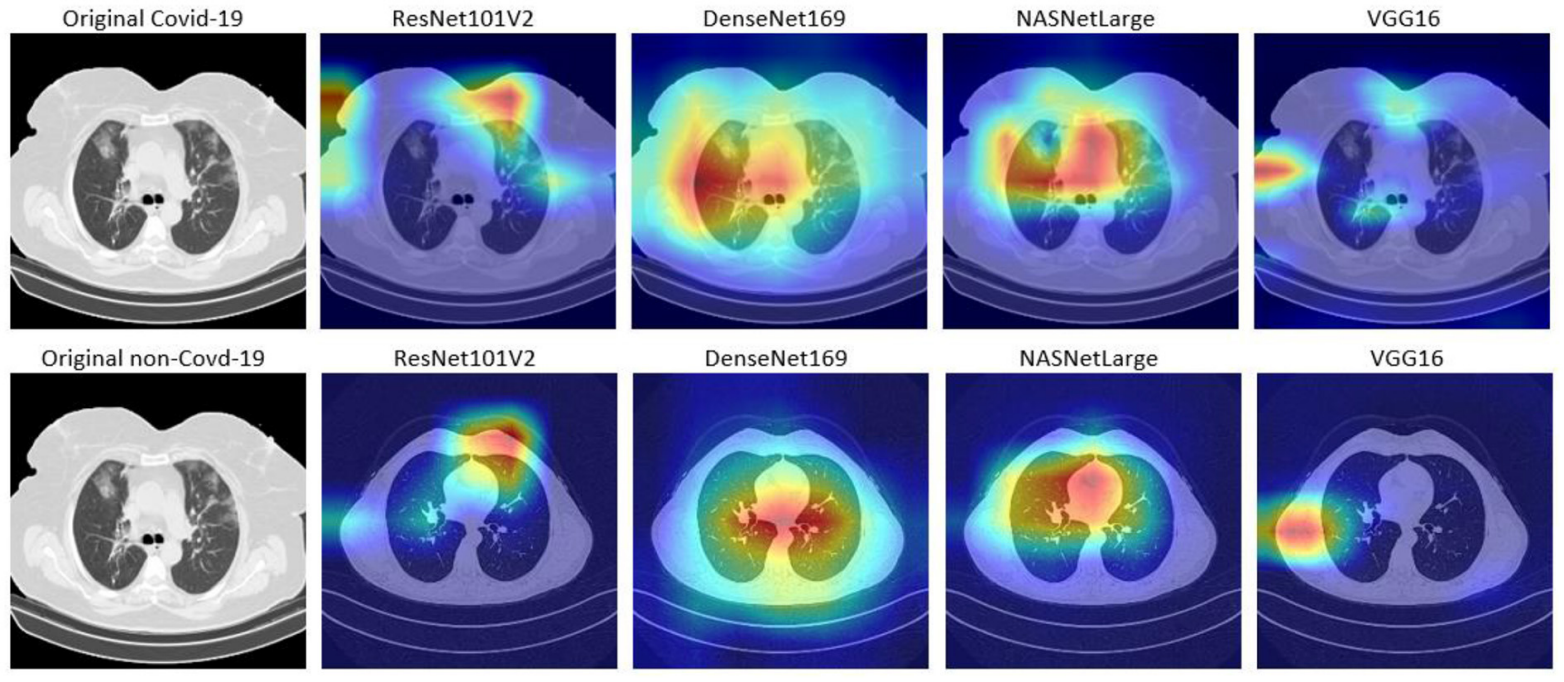
Localization maps for ResNet101V2, DenseNet169, NASNetLarge, VGG16 (without data augmentation).

**Figure 7:**
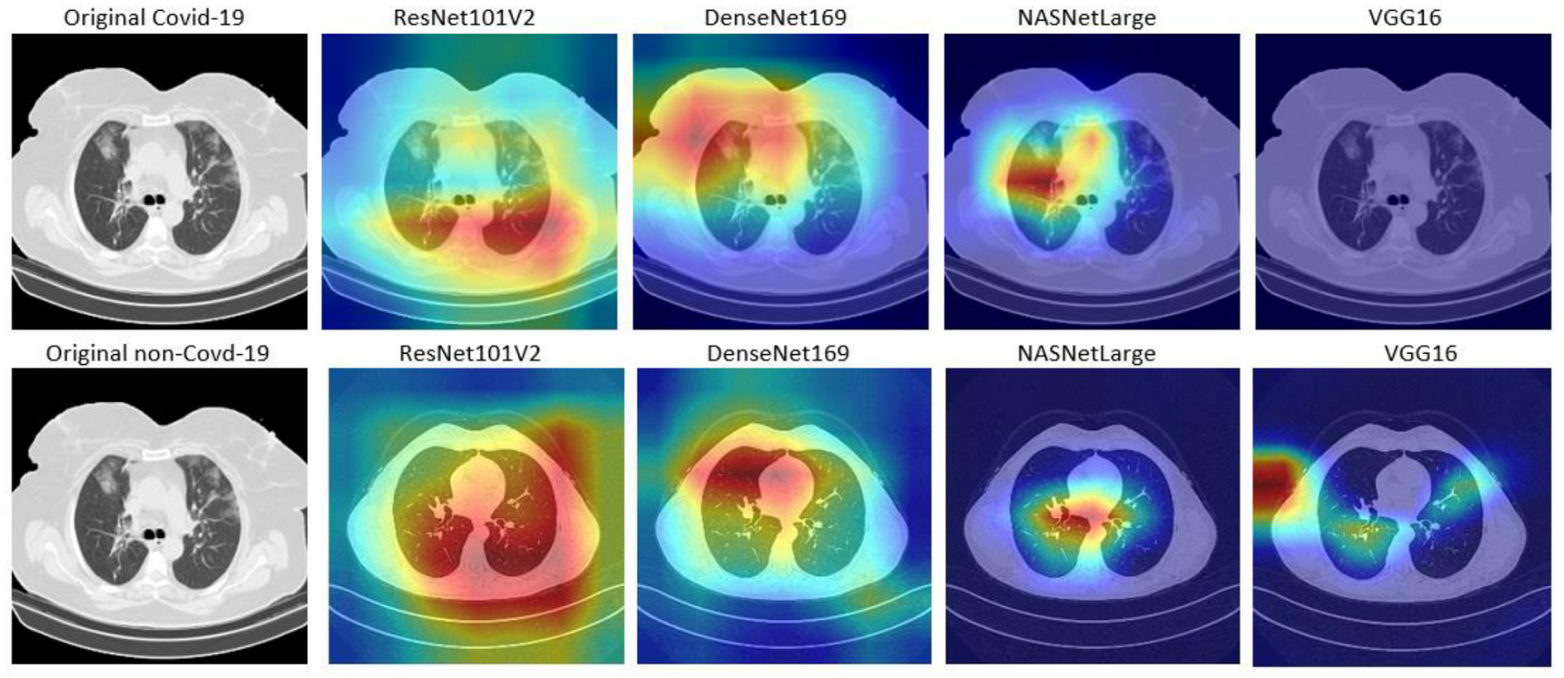
Localization maps for ResNet101V2, DenseNet169, NASNetLarge, VGG16 (with data augmentation).

In this study, Grad-CAM is used to visualize the patterns that were activated in the convolution layers of the models during the COVID-19 prediction. Figures 6, 7 shows the activations of four models, namely: VGG16, ResNet101V2, DenseNet169, and NASNetLarge. The four models are selected because they produced very good results in this study. Figure 6 shows the localization maps of the models that were not augmented, while Figure 7 shows the localization maps of the models that were augmented. The localization map is shown for both COVID-19 and non-COVID-19 images. As shown in Figure 6, DenseNet169 and NASNetLarge activated around similar regions of the COVID-19 and non-COVID-19 images. Also, Figure 7 shows that DenseNet169, NASNetLarge, and ResNet101V2 activated around similar regions. This activation behavior shows that the three models learned similar patterns that can be useful for diagnosing COVID-19 disease.

### Comparison with other techniques

The findings of this study are compared to those of three other studies: Ardakani et al. (2020), Horry et al. (2020), and Song et al. (2021). Song et al. (2021) introduced a DL-based system for CT diagnosis. They fine-tuned VGG16 and ResNet50 on 88 COVID-19 infected CT images and 86 non-CT images. ResNet50 produced the best accuracy of 93%. Ardakani et al. (2020) presented a performance analysis of 10 DL model, and the best performing model achieved a specificity, sensitivity and accuracy of 99.51, 100, and 99.02%, respectively. Horry et al. (2020) proposed a semi-automated DL technique for COVID-19 detection on X-Ray images. They evaluated their approach on 400 X-ray images (100 COVID-19 images), and five DL pre-trained models. The results showed that VGG19 achieved the best precision of 83%. As can be seen in Table 8, the fine-tuned models presented in this study outperformed the techniques proposed in the three studies. They produced better accuracy, sensitivity, and specificity than the compared techniques. The improved performance is most likely due to the diversity of the dataset, quality of the dataset, and the number of COVID-19 images used to train the models in this study. Most studies used between 60 and 600 COVID-19 images, however this study used 9,000 COVID-19 CT images and 5,000 non-COVID-19 CT images. This is higher than the number of images used in other studies (Ardakani et al., 2020; He et al., 2020; Horry et al., 2020; Loey et al., 2020; Song et al., 2021).

**Table 8:** Comparison with other techniques.

**Technique**	**VGG16**			**VGG19**			**ResNet50**			**InceptionV3**		**Xception**			**MobileNetV2**		
	**Acc**	**Sen**	**Spe**	**Acc**	**Sen**	**Spe**	**Acc**	**Sen**	**Spe**	**Acc**	**Sen**	**Acc**	**Sen**	**Spe**	**Acc**	**Sen**	**Spe**
Horry et al. (2020)	–	80	–	–	80	–	–	67	–	–	65	–	57	–	–	–	–
Song et al. (2021)	84	89	80	–	–	–	86	93	91	–	–	–	–	–	–	–	–
Ardakani et al. (2020)	83.33	80.39	86.27	85.29	92.16	78.43	94.12	90.20	100	–	–	99.02	98.04	100	92.16	97.06	87.25
Ours	99.643	99.436	100	99.750	99.887	99.51	99.929	100	99.81	98.429	97.517	99.929	99.887	100	97.929	100	94.36

*Acc, Accuracy; Sen, Sensitivity; Spe, Specificity*.

As shown in Tables 6, 7, the runtime of the pre-trained models is fairly high. This is because of the number of layers and parameters in each of the pre-trained models. The network with the least number of layers and parameters (i.e., MobileNetV2) has 53 layers and 3,538,984 parameters, which is still large. This shows that models with fewer number of layers will execute faster than the models examined in this study. Ramnarine (2021) developed a simple CNN architecture with two convolutional layer and two fully-connected layer. The model was trained on a dataset with 6,432 lung chest X-ray images, and it executed in 30 s. Moreover, the model achieved an accuracy of 100%.

### Training and validation loss

**Figure 8:**
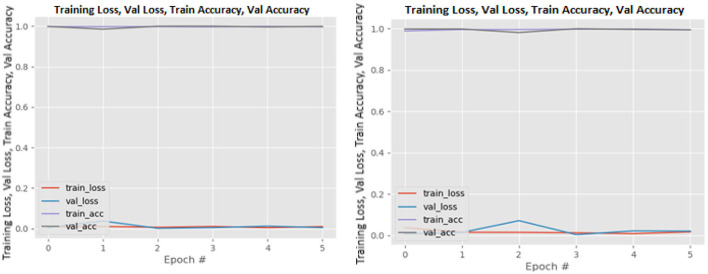
DenseNet121 without data augmentation (left image) and with data augmentation (right image).

**Figure 9:**
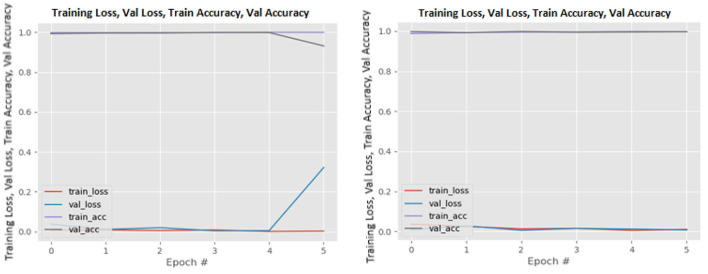
DenseNet169 without data augmentation (left image) and with data augmentation (right image).

**Figure 10:**
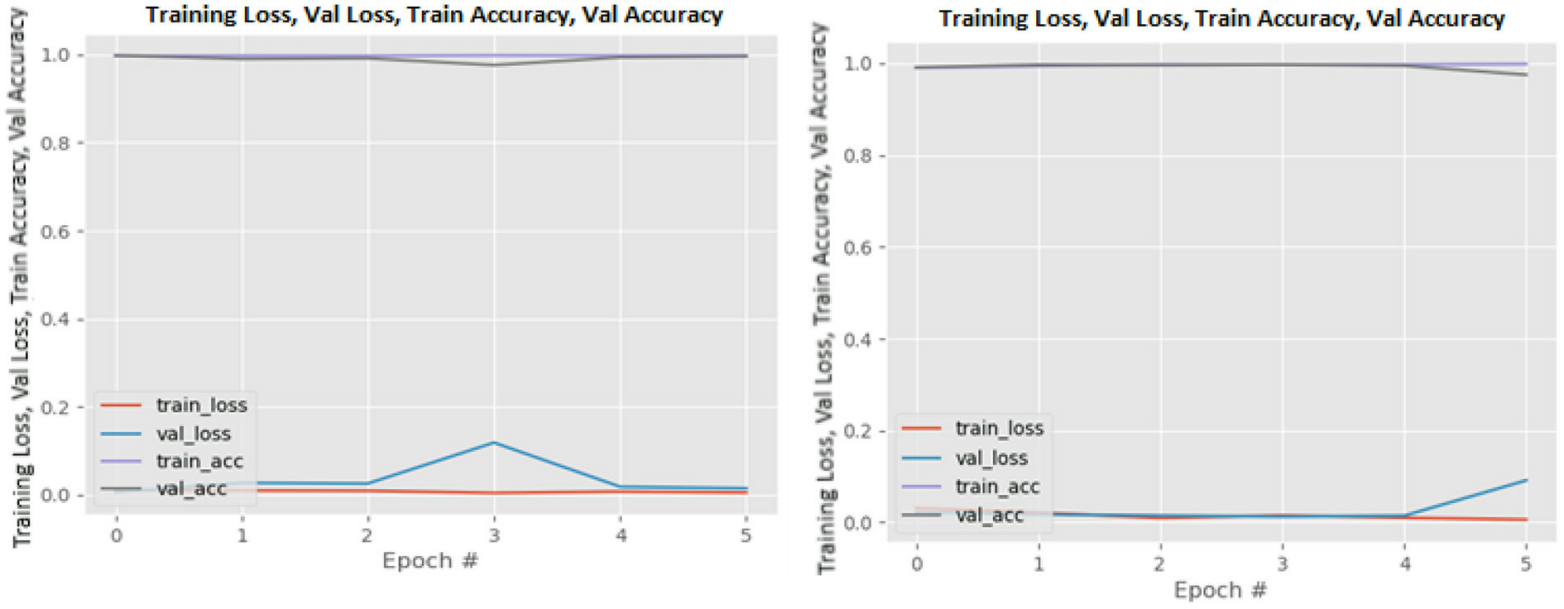
DenseNet201 without data augmentation (left image) and with data augmentation (right image).

**Figure 11:**
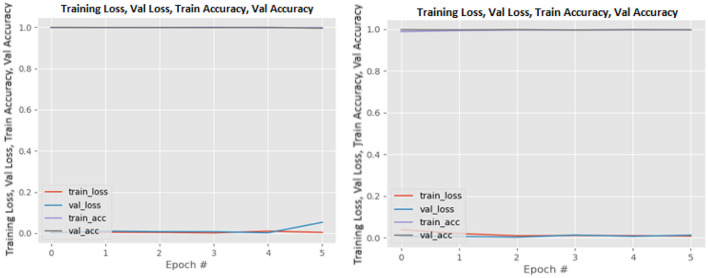
InceptionResNetV2 without data augmentation (left image) and with data augmentation (right image).

**Figure 12:**
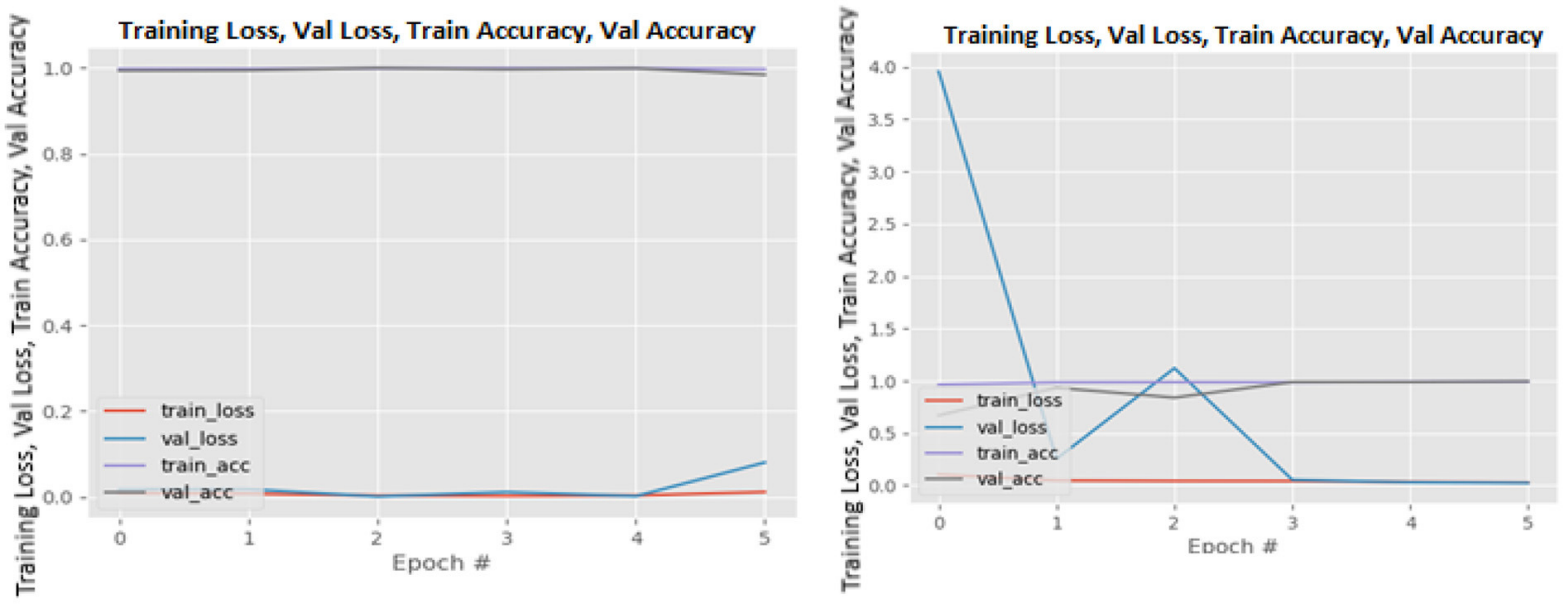
InceptionV3 without data augmentation (left image) and with data augmentation (right image).

**Figure 13:**
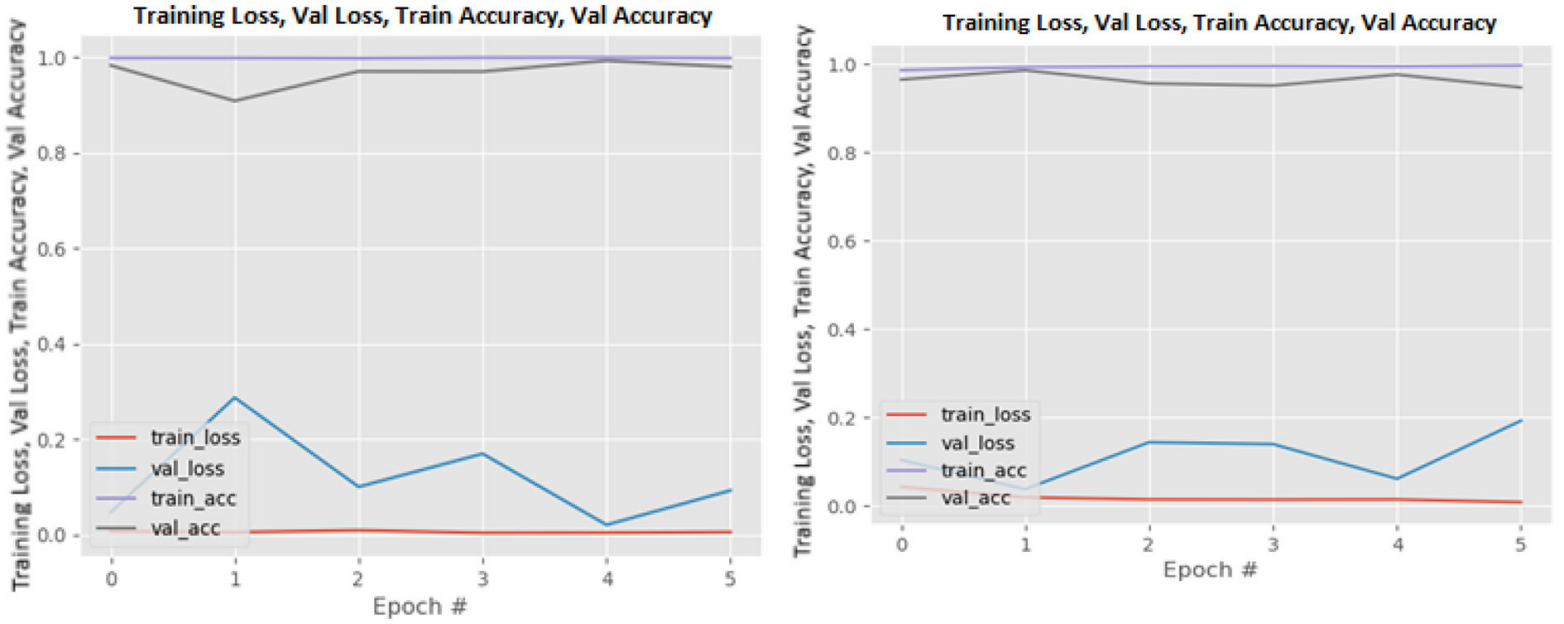
MobileNetV2 without data augmentation (left image) and with data augmentation (right image).

**Figure 14:**
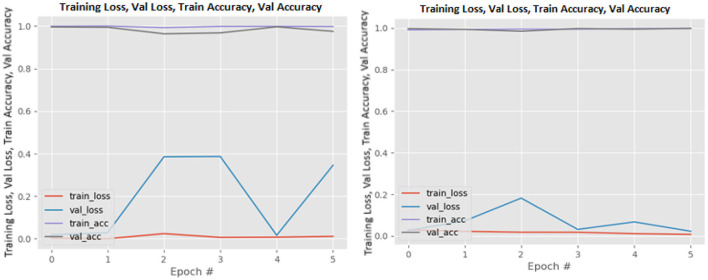
NASNetLarge without data augmentation (left image) and with data augmentation (right image).

**Figure 15:**
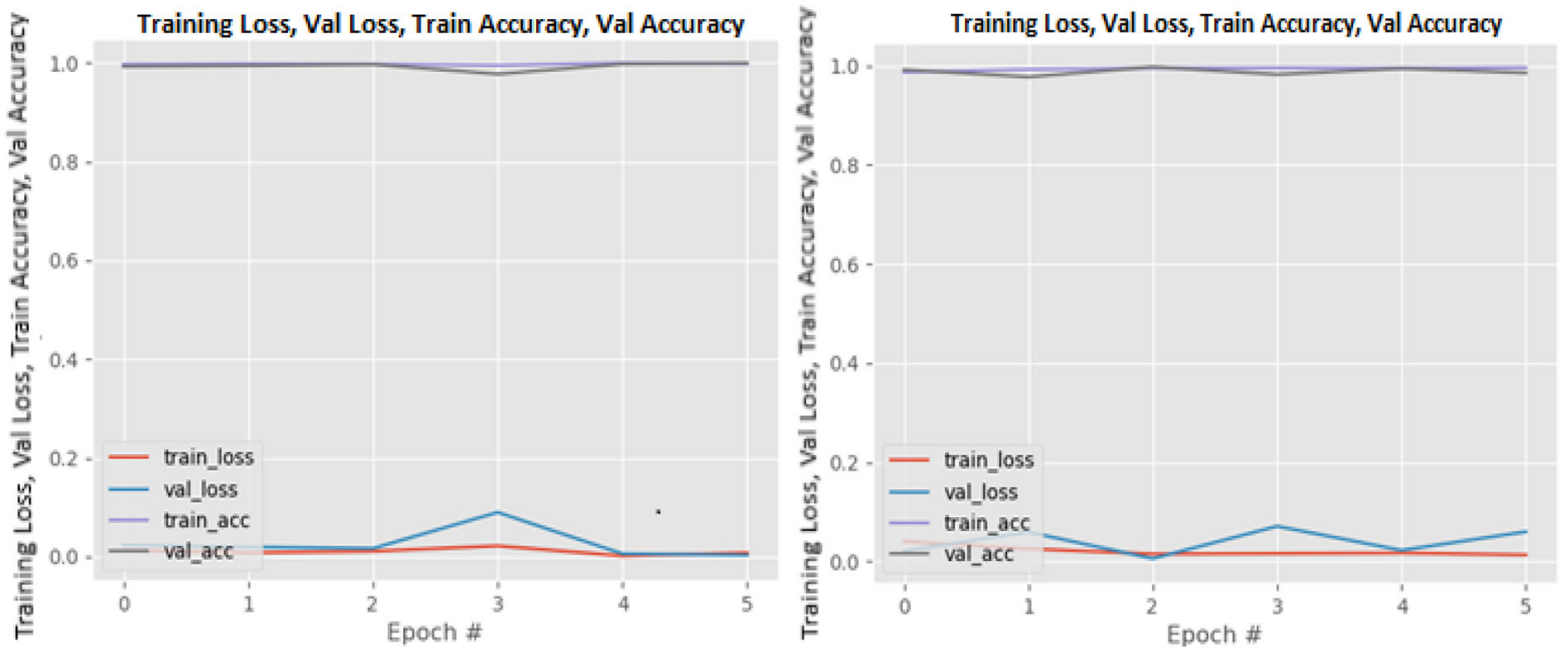
ResNet50 without data augmentation (left image) and with data augmentation (right image).

**Figure 16:**
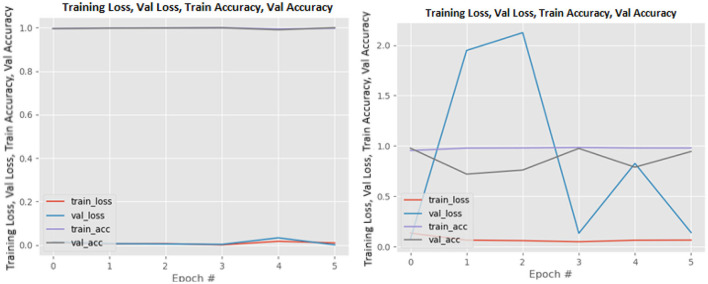
ResNet101V2 without data augmentation (left image) and with data augmentation (right image).

**Figure 17:**
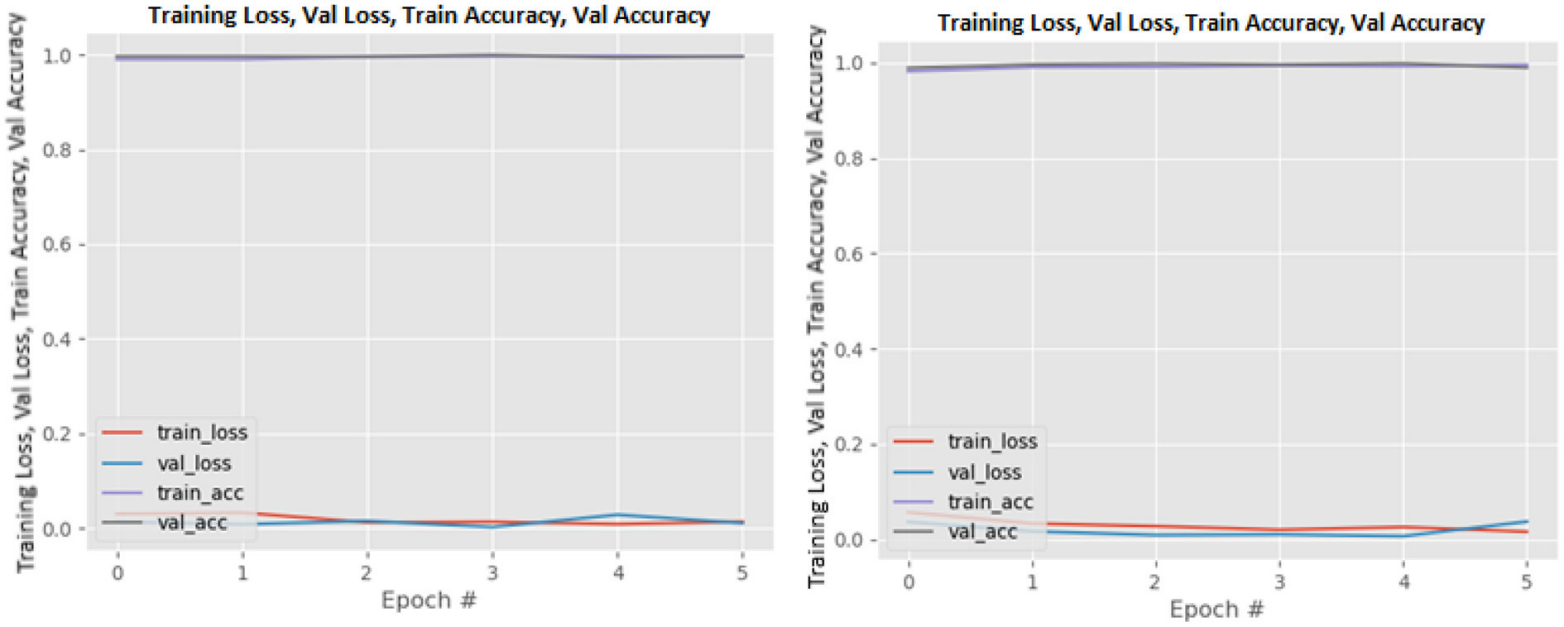
VGG16 without data augmentation (left image) and with data augmentation (right image).

**Figure 18:**
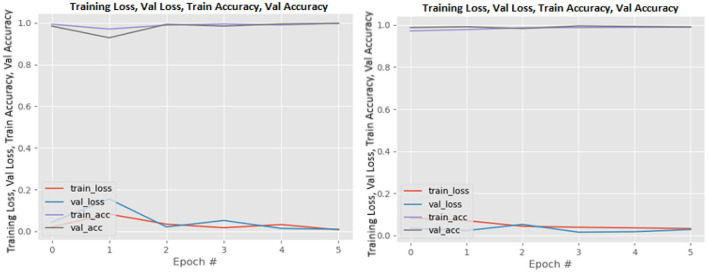
VGG19 without data augmentation (left image) and with data augmentation (right image).

**Figure 19:**
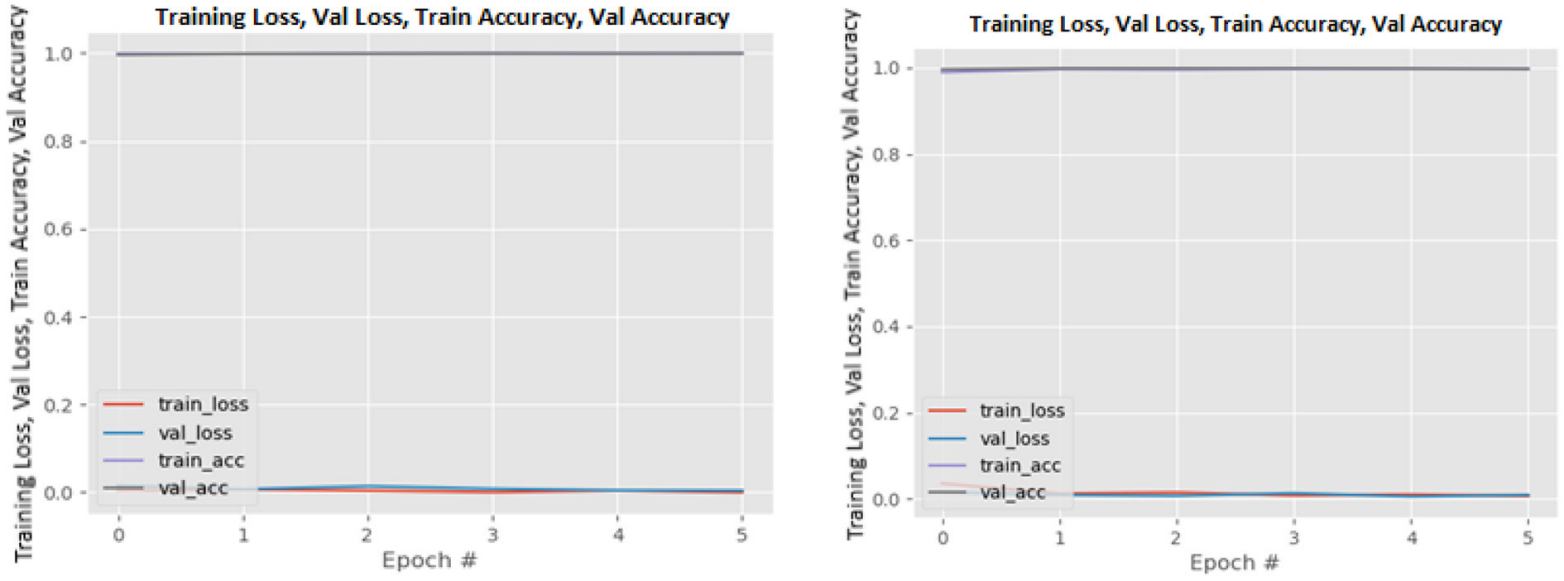
Xception without data augmentation (left image) and with data augmentation (right image).

Figures 8–19 show the training and validation loss of the proposed technique. The training and validation accuracies achieved by the proposed technique are also shown in the figures. The models were trained for two epochs during the hyper-parameter search step, as detailed in Section Performance of Pre-trained CNN Architecture With Data Augmentation. The best model was chosen at the end of hyper-parameter search stage and trained for another 6 epochs. The figures depict the models' performance during the second training stage. As seen in the figures, there is no major difference between the training and validation losses generated in each epoch. There is likewise no major difference in training and validation accuracy in each epoch. This demonstrates that the training models do not over-fit. It also shows that the models have good generalization ability.

## Summary

COVID-19 is a global pandemic that has killed millions of people worldwide. This study introduced state-of-the-art DL solutions for effectively diagnosing COVID-19 using CT images and 12 pre-trained DL models, including VGG16, VGG19, Xception, ResNet50, ResNet101V2, DenseNet169, InceptionV3, NASNetLarge, DenseNet201, MobileNetV2, InceptionResNetV2, and DenseNet121. Tables 6, 7 show the performances of the 12 DL models used in this study with their confusion matrix. As shown in the table, all the DL networks can effectively distinguish COVID-19 samples from non-COVID-19 samples with an accuracy within the range of 93.14–99.86%. The best classification accuracy was produced by NASNetLarge, followed by InceptionResNetV2 and DenseNet169. The accuracy of the three models is 99.86, 99.786, and 99.714%, respectively. DenseNet121 and VGG16 achieved the highest sensitivity, with both producing 99.944%. MobileNetV2 is the most efficient architecture in terms of computational efficiency, followed by InceptionV3 and ResNet50. All models were fine-tuned with data augmentation to increase their generalization performance. The results indicate that the models' sensitivity improved after data augmentation was implemented. This demonstrates the critical role of data augmentation in reducing overfitting and improving the generalization performance of the model.

The results of this study are compared to the results of three other studies. The comparison shows that the techniques presented in this study outperformed the three compared techniques. The dataset used in this dataset was validated in a study performed by the dataset authors (Gunraj et al., 2021). The validation was performed by two certified radiologists, and the validation shows that the decision-making behavior of the dataset is consistent with the interpretation of the radiologists. This demonstrates the potential of using DL in CT images as a non-invasive tool for automated COVID-19 diagnosis. We hope that this study will be valuable in improving the decisions and accuracy of medical practitioners when diagnosing COVID-19. This study can also aid future researchers in eliminating analysis repetition and determining the optimal network for their tasks. As future studies, researchers can investigate more efficient DL models (such as fix-efficientNet) and present a performance analysis of the models. Moreover, researchers can develop an ensemble model of pre-trained networks and evaluate their performance.

## Data availability statement

The original contributions presented in the study are included in the article/supplementary material, further inquiries can be directed to the corresponding author.

## Author contributions

Both authors listed have made a substantial, direct, and intellectual contribution to the work and approved it for publication.

## Funding

This work was supported by the University of the Free State, South Africa.

## Conflict of interest

The authors declare that the research was conducted in the absence of any commercial or financial relationships that could be construed as a potential conflict of interest.

## Publisher's note

All claims expressed in this article are solely those of the authors and do not necessarily represent those of their affiliated organizations, or those of the publisher, the editors and the reviewers. Any product that may be evaluated in this article, or claim that may be made by its manufacturer, is not guaranteed or endorsed by the publisher.
